# Anesthetic Techniques and Postoperative Cognitive Dysfunction in Older Adults: Current Evidence and Perioperative Strategies

**DOI:** 10.3390/medicina62071214

**Published:** 2026-06-23

**Authors:** Harrie Toms John, Megha Ann Sebastian, Mariya Riya Francis, Klavio Pine, Cezar Cristian Mihai Moisa, Nicoleta Negrut, Anca Ferician

**Affiliations:** 1 Doctoral School of Biomedical Sciences, Faculty of Medicine and Pharmacy, University of Oradea, 410087 Oradea, Romania; harrie.tomsjohn@student.uoradea.ro (H.T.J.); pine.klavio@student.uoradea.ro (K.P.); moisa.cezarcristianmihai@student.uoradea.ro (C.C.M.M.); 2 Faculty of Medicine, Medical University—Pleven, 1, Saint Kliment Ohridski Street, 5800 Pleven, Bulgaria; 26riyafrancis@gmail.com; 3 Department of Psycho-Neuroscience and Recovery, Faculty of Medicine and Pharmacy, University of Oradea, 410073 Oradea, Romania; 4 Department of Medical Disciplines, Faculty of Medicine and Pharmacy, University of Oradea, 410073 Oradea, Romania; anca.ferician@uoradea.ro

**Keywords:** anesthesia, cognition, neuroinflammation, pain, neurodegenerative

## Abstract

*Background and Objectives:* With the rising number of geriatric surgical patients, postoperative cognitive dysfunction (POCD) has become a major concern, linked to impairments in memory, attention, and executive function. POCD increases morbidity, prolongs hospitalization, and diminishes quality of life. This review examines the mechanisms underlying POCD, with emphasis on neuroinflammation, blood–brain barrier (BBB) disruption, and oxidative stress, and evaluates the impact of anesthetic techniques on cognitive outcomes in the elderly. *Materials and Methods:* This narrative review used a targeted literature search to identify relevant clinical, translational, and mechanistic evidence on POCD in older surgical patients. The evidence was synthesized qualitatively, with attention to heterogeneity in study populations, anesthetic techniques, cognitive assessment methods, and follow-up duration. *Results:* Neuroinflammation, BBB compromise, oxidative stress, perioperative stress responses, and patient vulnerability appear to contribute to POCD. Evidence comparing anesthetic techniques remains heterogeneous. Some studies suggest associations between general anesthesia, volatile agents, and early postoperative cognitive changes, whereas other comparative and randomized studies do not demonstrate consistent long-term cognitive differences between general, regional, neuraxial, volatile, and intravenous anesthetic approaches. Regional and neuraxial techniques may reduce anesthetic or opioid exposure in selected patients, but they should not be interpreted as definitively superior for POCD prevention. Adjunctive and multimodal strategies, including dexmedetomidine and non-opioid analgesics, show potential benefits, although evidence remains variable. *Conclusions:* Individualized anesthetic planning, early risk stratification, avoidance of excessive anesthetic depth, hemodynamic optimization, multimodal analgesia, and postoperative recovery strategies may help reduce modifiable contributors to POCD. Current evidence does not support a definitive hierarchy of anesthetic techniques for preventing POCD, and further high-quality studies are needed.

## 1. Introduction

With continuous advancements in the medical field and a growing global emphasis on healthier lifestyles, life expectancy has risen considerably [1]. As a result, surgical procedures on elderly patients become more prevalent due to the growing geriatric population. The unique perioperative challenges of elderly patients stem from physiological aging properties combined with multiple medical conditions and increased susceptibility to dangerous outcomes [2].

Postoperative cognitive dysfunction (POCD) has emerged as a major problem because it affects many patients and produces serious clinical effects [3]. Postoperative cognitive dysfunction impacts multiple mental faculties through disturbances in memory function, attention span, alertness levels, mood control, circadian patterns, and executive control abilities. Epidemiological research shows that cognitive impairment affects 40% of older adults after surgical hospital discharge, while 12.7% maintain these deficits three months after discharge [4]. The neurological damage results in extra morbidity and prolonged hospital stays along with reduced life quality while increasing mortality rates for long durations. The development of depression and anxiety becomes more likely in patients who have POCD, which continues to deteriorate their overall health outcome.

The pathophysiology of postoperative cognitive dysfunction remains undefined, yet recent studies indicate that surgical and anesthetic procedures trigger neuroinflammation as their primary mechanism. This developmental sequence includes the activation of inflammatory mediators together with blood–brain barrier (BBB) breakdown, synaptic plasticity damage, and neuronal cell death [5,6]. Cognitive decline risk increases for elderly patients because age-related vulnerabilities weaken their BBB resistance during these disruptions. Anesthetic agents create reactive oxygen species, which cause neuronal damage through oxidative stress, thus adding to POCD development [7].

This study aims to delve into the underlying pathophysiological mechanisms of POCD, drawing upon the latest research to explore the pathways involved in cognitive impairment associated with various anesthesia and analgesia techniques. We also seek to identify contributing factors specific to the elderly population. By synthesizing the existing literature, this work aspires to enhance anesthetists’ understanding of the cognitive impacts of different anesthetic practices. Ultimately, we aim to raise awareness about the importance of tailored anesthetic strategies, with the aim of enhancing postoperative cognitive recovery and overall quality of life in older patients.

## 2. Materials and Methods

This article is a narrative review of current evidence on postoperative cognitive dysfunction (POCD) in older surgical patients, with emphasis on pathophysiological mechanisms, perioperative risk factors, anesthetic techniques, and adjunctive strategies.

A targeted literature search was conducted in PubMed, Scopus, Web of Science, and Google Scholar for studies published between 2000 and 2026. Search terms included “postoperative cognitive dysfunction,” “POCD,” “postoperative neurocognitive disorders,” “geriatric surgery,” “neuroinflammation,” “oxidative stress,” “blood–brain barrier disruption,” “general anesthesia,” “regional anesthesia,” “neuraxial anesthesia,” “dexmedetomidine,” and “non-opioid analgesia.”

Because this was a narrative review rather than a systematic review or meta-analysis, no formal protocol registration, PRISMA flow diagram, risk-of-bias assessment, or quantitative synthesis was performed. Relevant original studies, clinical trials, systematic reviews, meta-analyses, and mechanistic studies were selected based on their relevance to POCD, anesthesia, and geriatric perioperative care. The evidence was synthesized qualitatively, with attention to heterogeneity in study populations, surgical procedures, anesthetic approaches, cognitive assessment methods, and follow-up duration.

As this review did not involve direct human or animal experimentation, ethical approval and informed consent were not required.

## 3. Pathophysiology of POCD in Geriatric Patients

### 3.1. Neuroinflammatory Mechanisms and Their Role in Cognitive Decline

Inflammation is a natural response to any systemic injury, infection, or toxic insult. Neuroinflammation specifically points to those inflammatory responses occurring in the central nervous system (CNS), mediated by activation of glial cells (microglia and astrocytes), infiltrating peripheral immune cells, and endothelial cells [5]. The activation of microglia and astrocytes leads to the secretion of pro-inflammatory cytokines, chemokines, reactive oxygen species, and secondary signaling molecules, which may cause a disruption of the BBB, impairing synaptic plasticity, leading to neuronal apoptosis [6]. All these processes together contribute to cognitive dysfunction.

The neuroinflammatory response is widely regarded as a central enabler of POCD. Invasive procedures like surgery trigger systemic inflammation, which crosses the BBB, leading to the activation of microglia and astrocytes in the brain. This activation subsequently promotes the secretion of pro-inflammatory cytokines, including tumor necrosis factor-alpha (TNF-α) and interleukins such as IL-1β, IL-6, and IL-2, and other mediators that lead to the disruption of neuronal pathways, ultimately impairing cognitive processes [8].

A systematic review highlighted the dual roles of neuroinflammation, in which, during the acute phase, the neuroinflammation showed to be neuro-protective but detrimental when it was prolonged for excessive periods, progressing to synaptic dysfunction and neuronal loss [9].

There is an overlap between Alzheimer’s disease (AD) and POCD, suggesting shared underlying mechanisms, including neuroinflammation. Studies in AD have demonstrated that neuroinflammation contributes to the buildup of amyloid-beta plaques and tau-associated neurofibrillary tangles, thereby exacerbating cognitive impairment [10]. The pleiotropic effects of neuroinflammation observed in AD, which encompass both protective and neurodegenerative roles, may also be relevant in the pathophysiology of POCD [9].

Chronic alcohol consumption has been associated with the buildup of iron in the brain, triggering microglial activation. The process then stimulates the production of pro-inflammatory cytokines and reactive oxygen species (ROS), leading to BBB disruption and neuronal damage [10]. The resulting chronic inflammation disrupts synaptic plasticity, memory, and attention, further accelerating neurodegeneration. This highlights the role of external factors in contributing to neuroinflammatory mechanisms and cognitive decline.

Diabetes mellitus is another condition that is associated with neuroinflammation. Insulin resistance, hyperglycemia, and altered insulin signaling contribute to oxidative stress and chronic inflammation. Oxidative stress is a major contributor to neuronal degeneration. Elevated levels of pro-inflammatory cytokines like TNF-α and IL-6 have been widely observed in diabetic patients who have had cognitive impairment [11].

Surgical trauma promptly induces extreme systemic and neuroinflammatory responses with an instant rise in inflammatory biomarkers, significantly seen in blood and in cerebrospinal fluid (CSF). A study in Danielson et al. (2020) revealed that the association between the intensity and duration of this inflammatory response are very closely associated with long-term cognitive outcome post-op [12]. Patients with elevated inflammatory markers in the CSF showed a greater degree of cognitive decline at three months post-op [12].

Neuroinflammation contributes to cognitive decline through several primary mechanisms. One key factor is the disruption of the BBB, where prolonged inflammation leads to weakening of the BBB, allowing the passage of circulating immune cells to infiltrate the CNS, further exacerbating inflammation [10]. Additionally, synaptic dysfunction and neuronal loss occur as a result of pro-inflammatory cytokines interfering with synaptic transmission, promoting neuronal apoptosis, leading to impaired cognitive function [6]. Another crucial mechanism is oxidative stress, where excessive production of ROS damages neuronal signaling pathways, accelerating neurodegeneration [11]. The accumulation of pathological proteins, like amyloid-beta and tau, intensifies neuroinflammation, creating a vicious cycle and further exacerbating cognitive decline [9].

To combat neuroinflammation and its impacts on cognitive decline, several strategies are being explored. Targeting inflammatory pathways with anti-inflammatory agents like inflammatory inhibitors may help reduce neuroinflammation and its cognitive effects. However, their effectiveness in pre and post op elderly patients requires further research. Another promising approach is early biomarker identification, where measuring inflammatory markers like TNF-6 in the blood and cerebrospinal fluid may help identify patients at risk for post-op cognitive dysfunction.

Pre-rehabilitation strategies include cognitive training, physical activity, and nutritional optimization, which may play a crucial role in improving post-surgical outcomes by lowering baseline inflammation. Additionally, the role of inflammatory genes needs further study, as research suggests the cytokine-encoding genes may actively promote cognitive decline in older adults. However, due to study limitations, the potential for false positives remains uncertain [13].

Lastly, co-morbidities and their role in POCD cannot be overlooked. Inflammation and disease progression are clearly influenced by underlying health conditions, but studying this link demands transgenic models in order to avoid oversimplification of the complex nature of aging and the interplay of multiple co-existing diseases [14].

Table 1 summarizes the major triggers, mechanisms, and cognitive effects associated with neuroinflammation-related cognitive impairment.

### 3.2. BBB Disruption During Surgery and Anesthesia

The blood–brain barrier (BBB) is a highly specialized semipermeable structure that is critical for maintaining CNS homeostasis by the tight regulation of the exchange of molecules and cells between the CNS and bloodstream. This protective structure is crucial to prevent harmful substances from entering the parenchyma of the brain. However, disruptions in the BBB during anesthesia and surgery, particularly in geriatric patients, are increasingly implicated in POCD.

The BBB acts as a checkpoint in the maintenance of the CNS environment. Any insult to this structure leads to brain dysfunction promoting to cognitive decline. In geriatric patients, age-related changes to the BBB make its integrity more vulnerable to damage during surgical and anesthetic interventions [15,16].

Anesthesia and surgery trigger systemic and localized neuroinflammation, often mediated through the disruption of the BBB. For instance, Propofol, an intravenous anesthetic, has been demonstrated to enhance the production of inflammatory mediators, including IL-1β, IL-8, and monocyte chemoattractant protein-1 (MCP-1), which enhances BBB permeability [17]. This inflammatory cascade predisposes patients to POCD [18].

Experimental animal model studies showed that older subjects experienced greater and prolonged BBB disruptions compared to the younger ones. Hu et al. (2025) demonstrated BBB damages that are age-dependent, where elderly mice showed more cognitive defects and BBB leakage due to impaired tight junctions and enhanced transcellular support [19].

To reduce the risk of POCD, a more personalized approach to patient care is essential. Patient risk assessment should include monitoring biomarkers linked to the BBB dysfunction, which helps in the identification of those at higher risk. Incorporating comprehensive geriatric assessments into perioperative care could further refine risk prediction to improve patient outcomes.

Another key consideration is tailored anesthetic strategies. Research suggests that certain anesthetic techniques can significantly impact the integrity of the BBB [17]. Coordinated selection and the usage of multimodal anesthesia approaches may help in minimizing inflammatory responses, hence reducing the risk of BBB disruption. Longterm follow up is also crucial as cognitive decline can develop gradually. Extending post-operative cognitive surveillance in elderly patients beyond the initial recovery phase may allow for the early detection and management of dementia related risks.

Moving forward, mechanistic research on BBB disruption is needed to better understand the molecular changes that occur during surgery and anesthesia. Specifically, investigating tight junctions, oxidative stress mediators, and pro-inflammatory cytokines contributing to BBB vulnerability will lead to more targeted interventions. Additionally, studying age-specific mechanisms will help determine how aging influences BBB integrity, paving the way for a better approach for elderly patients.

A particularly exciting area of research is the development of personalized anesthetic protocols based on molecular genetics and clinical risk factors. By tailoring anesthesia to an individual’s unique biologic profile, we may be able to protect the BBB more effectively, ultimately reducing the risk of cognitive complications post-surgery.

### 3.3. Oxidative Stress and Its Contribution to Neuronal Damage

Oxidative stress results from an imbalance between the production of reactive oxygen species (ROS) and reactive nitrogen species (RNS), and the capacity of the antioxidant system to neutralize them [7]. Cellular respiration is a primary source of ROS and RNS production, but excess production of these contributes to cellular dysfunction and death [20].

One major source of oxidative stress is mitochondrial dysfunction, where disruptions in the electron transport chain lead to excessive production of ROS. This oxidative overload leads to neuronal damage and elevated mitochondrial calcium influx, further worsening excitotoxic neuronal death, an essential driver of neurodegeneration [21,22].

Another key contributor is NADPH oxidase, an enzymatic complex that substantially increases ROS production, especially following acute neurological events such as stroke or traumatic brain injury. Studies show that inhibiting NADPH oxidase pharmacologically could limit neuronal damage, highlighting its role in neuroprotection [23]. However, chronic deregulation of NADPH oxidase has been linked to neurodegeneration, aging, and cardiovascular disease, suggesting it to be a critical target for further research [24].

Molecular damage induced by excess ROS causes damage in the brain. One harmful process is lipid peroxidation, where the ROS attack the lipid membrane of cells, generating highly reactive lipid peroxidation-derived aldehydes, including 4-HNE. These compounds further compromise the integrity of the membrane, damaging proteins and even inducing DNA mutations, further promoting cellular dysfunction [7].

Additionally, oxidative stress contributes to proteomic and genomic instability as the ROS-induced damage alters protein function and causes DNA strand breaks. This oxidative burden contributes to the weakening of neuronal resilience, exacerbating injury and increasing susceptibility to neurodegenerative conditions [25].

Chronic oxidative stress is linked to neuronal apoptosis and the onset of POCD in geriatric patients. Post-surgical interventions targeting oxidative stress could mitigate cognitive decline [18].

Nutritional antioxidants (e.g., polyphenols) reduce oxidative damage and offer neuroprotection in models of ischemic stroke [25].

**Figure 1:**
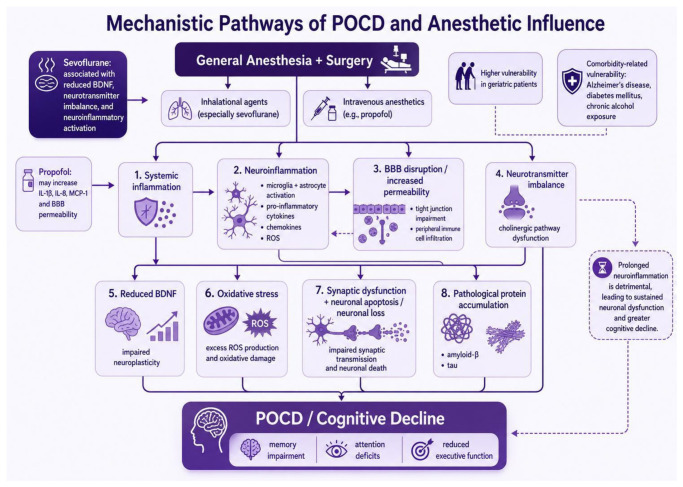
Mechanistic Pathways of POCD and Anesthetic Influence. POCD—Postoperative Cognitive Dysfunction; GA—General Anesthesia; BBB—Blood–Brain Barrier; BDNF—Brain-Derived Neurotrophic Factor; ROS—Reactive Oxygen Species; IL-1β—Interleukin-1 beta; IL-8—Interleukin-8; MCP-1—Monocyte Chemoattractant Protein-1; Amyloid- β—Amyloid Beta, CNS—Central Nervous System.

The complex interplay between surgical stress, anesthetic exposure, neuroinflammation, oxidative stress, BBB disruption, and neuronal dysfunction contributing to POCD is summarized in Figure 1.

## 4. Comparative Analysis of Different Anesthetic Techniques

### 4.1. General Anesthesia (GA) Versus Regional Anesthesia (RA) and Cognitive Outcomes

GA involves the use of intravenous or inhalational agents to induce unconsciousness, amnesia, and analgesia. While it is a cornerstone of modern surgical practice, GA has been linked with a higher risk of POCD in certain populations, especially geriatric patients [26].

Sevoflurane, a widely used inhalational agent in GA, has shown increasing evidence of being associated with POCD. Although its unique pharmacokinetics make it an effective anesthetic, decreased brain-derived neurotrophic factor (BDNF) concentration, pathological neurotransmitter imbalance, and activation of neuro-inflammatory pathways contribute to its risk of cognitive decline [26].

One major factor is neuroinflammation, as GA often triggers systemic inflammation that crosses the BBB and induces neuroinflammatory responses. This heightened inflammation exacerbates neuronal damage, increasing the risk of cognitive impairment [26]. Another key mechanism is the neurotransmitter imbalance, particularly in the cholinergic pathways, which are essential for memory function and cognitive processing. Disruptions in these pathways are believed to contribute to POCD [26].

Additionally, studies indicate that GA, especially with sevoflurane, is associated with reduced BDNF levels, a critical protein for neuroplasticity and cognitive function. This decline in BDNF is thought to be a key driver of sevoflurane- induced POCD alongside neuroinflammatory and neurotransmitter-related mechanisms [26].

Moving forward, research is focused on developing a combination of therapies that are focused on counteracting these effects and reducing sevoflurane’s impact on cognitive function.

Additionally, RA may help preserve neurotransmitter balance, minimizing the risk of neurotransmitter disruption, offering neuroprotection, and reducing the likelihood of POCD.

Despite its benefits, RA is not without limitations. It may be less suitable for certain surgeries, and individual patient factors such as comorbidities and surgical complexity influence their effectiveness.

#### Neuraxial Anesthesia and Cognitive Outcomes

Neuraxial anesthesia, including spinal and epidural anesthesia, represents an important regional anesthetic technique frequently employed in elderly surgical patients. By avoiding deep levels of general anesthesia and reducing perioperative opioid requirements, neuraxial approaches may contribute to improved early postoperative recovery and potentially reduce neurocognitive complications. In addition, neuraxial anesthesia may attenuate the physiological stress response to surgery, preserve hemodynamic stability, and facilitate earlier mobilization, all of which may indirectly support cognitive recovery in vulnerable geriatric populations [18].

Recent evidence comparing neuraxial and general anesthesia has yielded mixed findings regarding long-term cognitive outcomes. While some studies suggest lower rates of early postoperative cognitive impairment following regional techniques, contemporary randomized trials have generally demonstrated comparable rates of major postoperative neurocognitive outcomes between neuraxial and general anesthesia [27,28,29]. For example, a large randomized trial evaluating spinal versus general anesthesia in older adults undergoing hip surgery found no significant differences in major postoperative neurocognitive outcomes, suggesting that factors beyond anesthetic technique alone contribute substantially to postoperative cognitive trajectories [29].

These findings support the concept that postoperative cognitive dysfunction is multifactorial and influenced not only by anesthetic approach but also by patient characteristics, surgical stress, neuroinflammatory responses, baseline cognitive reserve, and overall perioperative management [3,18]. Consequently, the choice between neuraxial and general anesthesia should be individualized according to patient comorbidities, surgical requirements, and perioperative goals rather than based solely on expectations of cognitive benefit.

The key distinctions between general anesthesia, peripheral regional anesthesia, and neuraxial anesthesia in relation to postoperative cognitive outcomes, perioperative stress responses, hemodynamic considerations, and evidence limitations are summarized in Table 2.

### 4.2. Adjunctive and Multimodal Strategies

In elderly surgical patients, adjunctive anesthetic strategies and multimodal perioperative interventions have come into greater focus for their possible role in minimizing POCD. They all try to optimize the peri-operative neuroprotective strategies to include modulation of neuroinflammation, stabilizing cerebral metabolism, limiting anesthetic exposure, and improving recovery in the post-operative period.

Dexmedetomidine is a selective α2-adrenergic receptor agonist commonly used as a sedative supplement during RA and GA and has been shown to have neuroprotective effects in geriatric patients who have undergone surgery. The use of dexmedetomidine during the perioperative period has been reported to decrease the requirement for anesthetic use, improve cerebral oxygenation, decrease the metabolic stress, and decrease the serum concentrations of biomarkers of neuronal injury, including S100β and neuron-specific enolase (NSE) [30]. Patients receiving dexmedetomidine also have been found to have a decreased incidence of POCD during the early postoperative period when compared to control groups [30]. Possible mechanisms suggested are inhibition of sympathetic activation, decreased neuroinflammatory responses, maintenance of BBB integrity, and enhanced cerebral perfusion. However, current evidence has several limitations due to study methodologic heterogeneity, dosage schedules, cognitive assessment, and follow-up period. Further large-scale, multicenter trials are required to determine the optimal dosing strategy for dexmedetomidine in elderly patients and to evaluate its long-term neurocognitive effects in this population.

The choice of anesthetic maintenance may also have an impact on the cognitive outcomes after surgery. Total intravenous anesthesia (TIVA), particularly propofol-based regimens, has been evaluated as a potential alternative to inhalational anesthesia due to its anti-inflammatory properties as well as characteristics of a gentle recovery period. In a meta-analysis of 922 patients who underwent non-cardiac surgery, propofol-based TIVA was shown to improve Quality of Recovery-40 (QoR-40) scores measured on the surgical day compared with inhalational agents (sevoflurane) [31]. Other research has shown fewer pain scores and lower pain medication needs in TIVA patients [32]. The results indicate potential improvements in early postoperative recovery and patient comfort with TIVA.

But there is conflicting evidence between TIVA and inhalational anesthesia. Recent studies have failed to show any difference between pain scores, opioid use or cognitive function at 10 min or 48 h after surgery in patients receiving each anesthesia technique [33]. These conflicting results might be attributed to the different surgical techniques used, the different patient cohorts, the varying depth of anesthesia, the anesthetic used before and after surgery, and the different methods used for assessing cognitive function. Currently, there is no definitive evidence to prove that one anesthetic method is superior in preventing POCD.

In addition to pharmacologic therapies, multimodal perioperative strategies that combine non-pharmacologic therapies have been identified as potential means to enhance postoperative cognitive outcomes. They involve cognitive prehabilitation, physical exercise, sleep optimization, music therapy, environmental modification, non-invasive brain stimulation and transcutaneous electrical acupoint stimulation [34]. These strategies are intended to boost cognitive reserve, minimize reactions to perioperative stress, and promote recovery following surgery. There is emerging evidence for a synergistic effect of using non-pharmacologic interventions along with optimized anesthetic management for the reduction of POCD incidence and severity in the elderly [34]. However, there is still a need for standardised protocols and quality clinical trials to demonstrate the efficacy and feasibility of these multimodal strategies in everyday perioperative care.

Combined anesthetic approaches are increasingly utilized in geriatric surgical practice. The addition of regional or neuraxial anesthesia to general anesthesia may reduce intraoperative anesthetic requirements, decrease opioid consumption, improve postoperative analgesia, and attenuate the surgical stress response. These benefits may indirectly support cognitive recovery by limiting neuroinflammatory activation and facilitating earlier mobilization [18,30,31,32,33,34]. Although definitive evidence demonstrating a reduction in long-term POCD remains limited, combined anesthetic techniques represent an important component of individualized multimodal perioperative care for older adults [18,34]. Combined regional anesthesia with either volatile-based GA or propofol-based TIVA may facilitate anesthetic-sparing effects and enhanced multimodal recovery pathways in elderly patients [30,31,32].

Overall, adjunctive and multimodal perioperative strategies represent an evolving area of interest in geriatric anesthesia. While several interventions demonstrate promising neuroprotective potential, the current literature remains heterogeneous, and further research is needed to clarify their long-term clinical benefits and their potential role in preventing POCD.

## 5. Preoperative Risk Factors for POCD

### 5.1. Age, Comorbidities, and Baseline Cognitive Function

The development of POCD has been linked to multiple factors, including patient-related characteristics, surgical variables, and anesthetic techniques. Among these, advancing age and diabetes mellitus appear to be among the most consistently reported risk factors. Based on the available literature, POCD risk factors can generally be classified into patient-related, surgery-related, anesthesia-related, and other contributing factors (Table 3).

### 5.2. Pre-Existing Neurodegenerative Conditions and Their Exacerbation Post-Surgery

Pre-existing neurodegenerative disorders are recognized as important contributors to the development of POCD in elderly patients. Multiple perioperative factors, including surgical stress, anesthetic exposure, medication use, neuroinflammatory responses, pathogenic protein accumulation, neurotransmitter dysregulation, chronic psychological stress, and vitamin D deficiency, may further amplify this risk [8]. Moreover, pre-existing conditions such as Alzheimer’s disease and mild cognitive impairment (MCI) can be exacerbated by the physiological and inflammatory burden associated with surgery and anesthesia. Surgery-induced inflammation can alter microglial morphology by releasing pro-inflammatory cytokines, which further damage the neuron structure, leading to cognitive impairment. The interaction of anesthetic agents with multiple medications taken by patients with neurodegenerative conditions also has a higher potential to develop POCD.

A comprehensive understanding of these risk factors enables healthcare professionals to perform more effective preoperative and postoperative assessments, facilitating individualized patient care and the early implementation of preventive strategies against POCD. Several potentially modifiable factors, including lifestyle optimization, correction of vitamin D deficiency, appropriate perioperative glycemic control, and the selection of safer anesthetic approaches, may help reduce the incidence of POCD in elderly patients. However, current evidence concerning the contribution of genetic predisposition in the development of POCD is limited, underscoring the need for further large-scale investigations to clarify these associations [8].

## 6. Intraoperative Considerations

### 6.1. Depth of Anesthesia Monitoring and Its Effects on POCD

**Figure 2:**
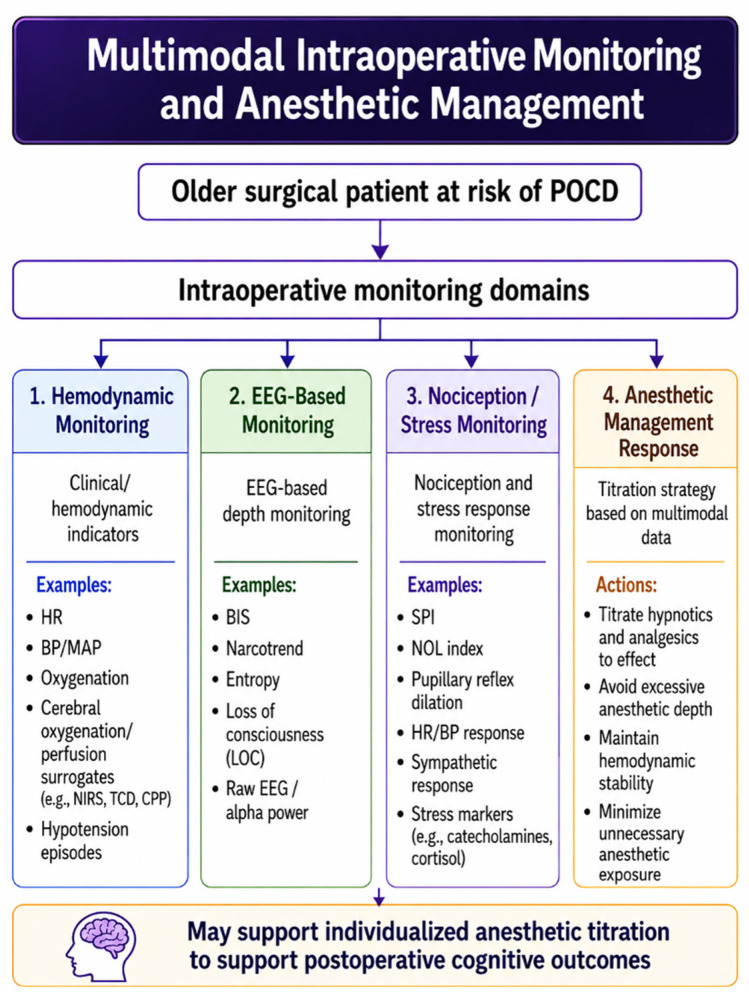
Multimodal intraoperative monitoring domains for individualized anesthetic management in older surgical patients at risk of postoperative cognitive dysfunction. Clinical and hemodynamic indicators, EEG-based depth monitoring, nociception/stress monitoring, and anesthetic management responses may help guide titration of hypnotics, analgesics, and vasoactive support. These strategies may assist in avoiding excessive anesthetic depth, hemodynamic instability, and unnecessary anesthetic exposure; however, their effects on postoperative cognitive outcomes remain uncertain and may vary according to patient characteristics, surgical context, anesthetic technique, and perioperative management. BIS, bispectral index; BP, blood pressure; CPP, cerebral perfusion pressure; EEG, electroencephalography; HR, heart rate; LOC, loss of consciousness; MAP, mean arterial pressure; NIRS, near-infrared spectroscopy; NOL, nociception level; POCD, postoperative cognitive dysfunction; SPI, surgical pleth index; TCD, transcranial Doppler.

Careful assessment and optimization of anesthetic depth may have a significant role in reducing the risk of POCD in elderly patients. Intraoperative monitoring can be achieved through hemodynamic parameters such as heart rate and mean arterial pressure (MAP), and through advanced neurophysiological monitoring techniques evaluating cerebral activity [49]. Commonly utilized modalities include electroencephalography (EEG), Bispectral Index (BIS), alpha power analysis, entropy-derived EEG parameters such as Lempel–Ziv complexity (LZc) and permutation entropy (PE), and the Narcotrend index, as well as the Index of Consciousness (IoC) [50,51,52]. The principal intraoperative monitoring domains that may guide individualized anesthetic management are summarized in Figure 2.

Some studies suggest that processed EEG-guided or multimodal monitoring may be associated with improved postoperative cognitive outcomes in selected elderly surgical populations; however, evidence remains heterogeneous and does not establish definitive POCD prevention. For example, Narcotrend-guided anesthesia has been associated with improved postoperative cognitive outcomes in some elderly surgical cohorts undergoing gastrointestinal tumor surgery [49,50]. Similarly, BIS monitoring remains an important component of individualized anesthetic management, allowing more precise titration of anesthetic agents while minimizing excessive anesthetic exposure [50]. IoC monitoring, a more recent EEG-based modality assessing both sedation and analgesia, may contribute to attenuating intraoperative stress responses and limiting peripheral and central neuroinflammatory injury [50,52]. In addition, entropy monitoring combined with the Surgical Pleth Index (SPI) has demonstrated potential benefits in reducing POCD following emergency surgical procedures [53].

The incidence of POCD may also be influenced by the anesthetic agents administered. Evidence from patients undergoing coronary artery bypass grafting (CABG) indicates that propofol-based total intravenous anesthesia (TIVA) may be associated with a lower incidence of POCD than sevoflurane-based volatile anesthesia [54,55]. In geriatric patients undergoing laparoscopic inguinal hernia repair, reducing the propofol infusion rate did not significantly decrease the incidence of early POCD; however, it reduced the total induction dose and the requirement for vasoactive agents, contributing to greater hemodynamic stability [56]. Current expert recommendations emphasize individualized anesthetic management, multimodal perioperative strategies, and postoperative brain-health optimization to reduce perioperative neurocognitive disorders [57].

Overall, individualized monitoring may help guide anesthetic titration and avoid excessive anesthetic depth or hemodynamic instability, but its independent effect on postoperative cognitive outcomes remains uncertain.

### 6.2. Neuroprotective Strategies During Surgery in the Elderly

Several neuroprotective strategies have been evaluated for their potential to reduce the incidence of POCD in elderly patients. Experimental and clinical studies have explored mechanisms such as matrix metalloproteinase-9 (MMP-9) inhibition, activation of sirtuin 1 (SIRT1), electroacupuncture, normobaric hyperoxia preconditioning, and upregulation of triggering receptor expressed on myeloid cells 2 (TREM2), alongside the use of pharmacological agents including metformin, varenicline, dexmedetomidine, and amantadine, all of which demonstrated varying degrees of neuroprotective and cognitive-preserving effects in perioperative settings [40,48,58,59,60,61,62,63,64]. Amantadine has shown promising effects in maintaining postoperative cognitive performance and may attenuate propofol-associated cognitive impairment [64].

Despite these encouraging findings, the long-term safety, efficacy, and clinical applicability of many of these strategies remain insufficiently established and warrant further investigation through larger prospective studies. In addition, current evidence regarding the influence of intraoperative hemodynamic stability and optimized oxygen delivery on POCD prevention in elderly patients remains limited, supporting the need for more focused studies on this topic.

### 6.3. Intraoperative Hemodynamic Instability and Postoperative Cognitive Dysfunction

In the context of the global aging trend, there has been a greater need for surgical therapeutic interventions in older individuals. Within said population, arterial hypertension represents a major source of intraoperative hemodynamic instability, often due to suboptimal pharmacological control. Besides arterial hypertension, a range of age-related physiological changes, such as advancement of atherosclerotic processes, impairment of endothelial function, and a decline in vascular elasticity, render elderly patients with hypertension particularly susceptible to high intraoperative fluctuations in blood pressure. This hemodynamic lability is closely linked with a greater risk of perioperative complications and exerts a detrimental impact on surgical outcomes, long-term prognosis, and postoperative outcomes [65]. Beyond the direct effects of blood pressure fluctuations on cerebral perfusion and neural function, accumulating evidence underscores the contributory role of circulating biochemical markers in the pathogenesis of postoperative cognitive impairment. Elevated serum alkaline phosphatase (ALP) levels have been recognized as a significant factor associated with the development of POCD. Notably, increased ALP levels have been identified as a predictor of cognitive decline at three months postoperatively. It could potentially reflect an underlying systemic inflammatory milieu implicated in the pathophysiological mechanism of POCD [38].

## 7. Postoperative Factors Influencing POCD

### 7.1. Pain Management and Its Cognitive Implications (Opioids vs. Non-Opioid Regimens)

Esketamine, the active S-enantiomer of ketamine, has gained attention as a promising non-opioid analgesic with multiple potential benefits in postoperative care. Administration of a single intraoperative dose of 0.5 mg/kg has been associated with reductions in postoperative pain, anxiety, and depressive symptoms [66]. In addition to its analgesic properties, esketamine has demonstrated anti-inflammatory effects through the attenuation of inflammatory cytokine release as well as the reduction of biomarkers associated with neuronal injury, suggesting a possible protective role against the development of POCD [66]. Moreover, esketamine appears to increase brain-derived neurotrophic factor (BDNF) expression, a neuroprotective mediator implicated in neuronal plasticity and cognitive recovery [66]. Owing to these combined analgesic, anti-inflammatory, and neuroprotective properties, esketamine may represent a valuable component of multimodal perioperative pain management strategies aimed at minimizing opioid exposure while supporting postoperative cognitive outcomes in elderly patients.

Dexmedetomidine, an α2-adrenergic receptor agonist, has emerged as a promising agent for lowering the incidence of postoperative cognitive dysfunction, especially in elderly patients. Several studies have demonstrated that dexmedetomidine, especially when combined with anesthetic agents such as sevoflurane, may significantly decrease the short-term occurrence of POCD in patients undergoing procedures including thoracic surgery [67]. These beneficial effects are likely related to its sedative, analgesic, and sympatholytic properties, which may reduce perioperative opioid requirements and consequently limit opioid-associated cognitive impairment.

The efficacy of dexmedetomidine appears to be affected by both the timing and clinical context of administration. While prolonged postoperative infusion did not significantly reduce postsurgical delirium in patients recovering from elective cardiac surgery, intraoperative administration has been associated with improved postoperative cognitive function outcomes in elderly patients undergoing abdominal surgery [67]. In addition, dexmedetomidine has demonstrated favorable outcomes when used as part of balanced anesthetic regimens. For example, a propofol–etomidate combination administered alongside dexmedetomidine has been proposed as an effective anesthetic strategy capable of maintaining adequate sedation and analgesia while decreasing the need for high-dose opioids and limiting their associated neurocognitive adverse effects [68].

Further evidence suggests that dexmedetomidine can decrease intraoperative requirements for agents such as remifentanil and propofol during minimally invasive procedures, including ureteroscopic holmium laser lithotripsy [69]. Moreover, its administration has been associated with decreases in postoperative emergence agitation, anxiety, and depressive symptoms among elderly patients, supporting its potential role in multimodal perioperative strategies aimed at preserving cognitive function and enhancing postoperative recovery [69].

Parecoxib, a selective cyclooxygenase-2 (COX-2) inhibitor, has also shown potential benefits in lowering POCD incidence and improving postoperative cognitive outcomes, including Mini-Mental State Examination (MMSE) scores [70]. Perioperative administration of parecoxib was associated with favorable cognitive outcomes in studies conducted within Chinese populations; however, further randomized controlled trials involving larger and more diverse populations are required before broad clinical applicability can be established [70].

Recent investigations additionally suggest that S-ketamine may represent a safe and effective adjunct for GA, with potential benefits extending beyond analgesia to include enhanced postoperative recovery and cognitive support [71]. In patients undergoing major procedures such as mastectomy, S-ketamine has been associated with improved pain control, emotional well-being, physical comfort, and early postoperative cognitive recovery. Notably, cognitive improvement observed on postoperative day one supports its potential role as a cognitively favorable analgesic option [71].

Effective perioperative pain management in elderly patients remains challenging, as optimal analgesia must be achieved while minimizing adverse neurocognitive effects. Chronic and acute painful conditions are highly prevalent in older adults, yet the complex interaction between pain, cognition, and analgesic therapies remains insufficiently understood [72]. Current evidence indicates that certain analgesics, particularly opioids, may contribute to cognitive decline in vulnerable elderly populations, emphasizing the importance of careful drug selection, individualized dosing, and multimodal analgesic approaches [72,73].

To further optimize patient-centered perioperative outcomes, future research should aim to develop comprehensive conceptual frameworks that assess both the direct effects of pain and the cognitive consequences of analgesic therapies. Such strategies may help maximize analgesic efficacy while minimizing postoperative neurocognitive complications in elderly surgical patients.

### 7.2. Sleep Disturbance and Postoperative Delirium

Perioperative sleep quality has emerged as an important factor influencing postoperative recovery and neurocognitive outcomes in surgical patients [74]. Several studies support the perioperative use of dexmedetomidine for improving postoperative sleep quality, particularly in elderly individuals undergoing major surgical procedures [75]. Nevertheless, the optimal dosing regimens and the overall impact of dexmedetomidine on postoperative sleep architecture and long-term cognitive outcomes remain insufficiently established, emphasizing the need for larger randomized controlled trials [75].

Non-pharmacological interventions have shown promise in improving sleep quality among critically ill patients. A systematic review indicated that earplugs and eye masks may be effective in enhancing sleep in ICU patients exposed to repeated nighttime assessments and environmental noise or light disturbances. These findings highlight the potential value of simple supportive measures as part of sleep-promoting strategies in perioperative and critical care practice [76].

Additional evidence suggests that low-dose dexmedetomidine supplementation in patient-controlled intravenous analgesia (PCIA) regimens containing sufentanil may improve the pain–sleep interaction cycle while further reducing the incidence of POCD [77]. In most studies, dexmedetomidine doses ranging from 2 to 5 μg were incorporated into analgesic protocols, contributing not only to improved analgesia but also to enhanced sleep quality and postoperative recovery [77].

Obstructive sleep apnea identifies a particularly vulnerable surgical population, given its association with increased postoperative risks such as cardiopulmonary complications, ICU transfer, delirium, pneumonia, bleeding events, and prolonged hospitalization [65,78]. The severity of obstructive sleep apnea appears to be an important modifier of postoperative risk, while preoperative identification and continuous positive airway pressure (CPAP) therapy may help reduce perioperative complications [65,78]. However, current evidence remains limited, and these findings should be interpreted cautiously until confirmed by larger prospective studies.

Complementary therapeutic approaches have also been investigated. For example, foot reflexology massage was evaluated for its potential impact on delirium incidence and postoperative sleep quality among patients undergoing cardiac surgery [74]. Although this intervention did not significantly improve sleep quality or reduce delirium incidence, it was associated with decreased postoperative pain intensity [74]. These findings further emphasize the complexity of delirium pathophysiology and highlight the need for more accurate predictive models and individualized preventive strategies.

**Figure 3:**
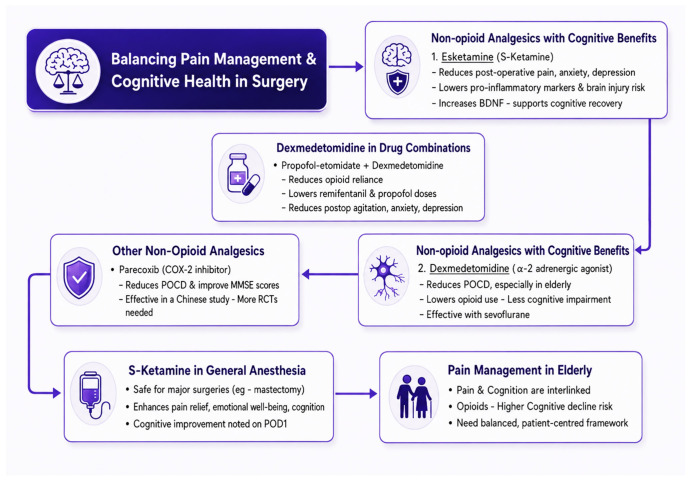
Balancing Pain Management and Cognitive Health in Surgery. POCD—Postoperative Cognitive Dysfunction; BDNF—Brain-Derived Neurotrophic Factor; MMSE—Mini-Mental State Examination; RCTs—Randomized Controlled Trials; POD1—Postoperative Day 1; COX-2—Cyclooxygenase-2; α-2—Alpha-2; eg—for example.

Collectively, the reviewed studies underline the importance of early rehabilitation and cognitive recovery interventions across several neurological conditions, including Alzheimer’s disease, traumatic brain injury, and stroke. Early cognitive rehabilitation programs, particularly in patients with early-stage dementia, have demonstrated the potential to slow cognitive decline, improve daily functioning, and enhance long-term quality of life. Moreover, individualized rehabilitation strategies tailored to patient-specific cognitive and functional needs may further optimize postoperative recovery and neurocognitive outcomes (Figure 3).

## 8. Conclusions

POCD represents a complex and multifactorial complication frequently encountered in elderly surgical patients. Its pathophysiology involves a combination of neuroinflammatory responses, BBB disruption, oxidative stress, perioperative cerebral hypoperfusion, and neurochemical alterations associated with surgical stress and anesthetic exposure. Given the growing number of surgical procedures performed in aging populations, a clearer understanding of these underlying mechanisms is essential for improving perioperative management and postoperative cognitive outcomes.

Current evidence suggests that anesthetic techniques may influence early postoperative cognitive outcomes, but findings remain heterogeneous and are affected by patient vulnerability, surgical stress, anesthetic depth, hemodynamic stability, perioperative inflammation, and differences in cognitive assessment methods. Some studies have reported associations between general anesthesia, volatile agents, or excessive anesthetic depth and early postoperative cognitive changes, whereas other comparative and randomized studies have not shown consistent long-term differences between general, regional, neuraxial, volatile, and intravenous anesthetic approaches. Regional and neuraxial techniques may reduce systemic anesthetic or opioid exposure in selected patients, but current evidence does not establish definitive superiority of any single anesthetic technique for preventing POCD. Adjunctive and multimodal strategies, including dexmedetomidine and non-opioid analgesic approaches, may support perioperative recovery, although their long-term cognitive benefits require further study.

Several patient-related factors appear to increase susceptibility to POCD, including advanced age, diabetes mellitus, baseline cognitive impairment, and pre-existing neurodegenerative disorders. Consequently, preventive perioperative strategies should focus not only on anesthetic selection but also on comprehensive patient assessment and individualized perioperative optimization. Intraoperative depth of anesthesia monitoring, maintenance of hemodynamic stability, multimodal anesthetic protocols, optimized postoperative analgesia, and interventions targeting sleep quality may all contribute to reducing postoperative cognitive decline in geriatric patients.

Future research should prioritize personalized perioperative care models that integrate biomarker-guided risk assessment, inflammatory and metabolomic profiling, individualized anesthetic strategies, optimized hemodynamic and respiratory management, patient-specific neuroprotective interventions, and long-term cognitive monitoring in elderly surgical patients. This may be particularly relevant in patients with recent or previous COVID-19 infection, in whom respiratory complications, systemic inflammation, hypoxemia, and prolonged postoperative recovery may increase vulnerability to neurocognitive decline [79,80,81]. Because postoperative cognitive outcomes are influenced by multiple perioperative factors, multidisciplinary strategies should aim to preserve cognitive function while improving overall recovery and quality of life in older surgical populations.

## Figures and Tables

**Table 1:** Pathways of Neuroinflammation and Cognitive Decline.

Trigger	Mechanism	Effect
Surgery	Systemic inflammation BBB disrupted Activation of microglia Release of cytokines	Cognitive impairment and POCD
Alzheimer’s disease	Accumulation of amyloid-beta and tau proteins Chronic neuroinflammation	Neurodegeneration
Diabetes mellitus	Hyperglycemia with insulin resistance Oxidative stress Overexpression of pro-inflammatory cytokines.	Cognitive decline and neuronal apoptosis
Chronic alcohol consumption	Accumulation of iron Microglial activation ROS production BBB disruption	Impaired memory Attention and decision making.

BBB—blood–brain barrier; POCD—postoperative cognitive dysfunction; ROS—reactive oxygen species.

**Table 2:** Comparison of general, peripheral regional, and neuraxial anesthesia in relation to postoperative cognitive outcomes in older adults.

Domain	General Anesthesia	Peripheral Regional Anesthesia	Neuraxial Anesthesia
Definition	Systemic anesthesia using intravenous and/or inhalational agents to produce unconsciousness, amnesia, and immobility.	Local anesthetic techniques targeting peripheral nerves, plexuses, or fascial planes.	A subtype of regional anesthesia involving spinal or epidural administration.
Cognitive outcome evidence	Some studies link GA, especially volatile exposure or excessive depth, with early cognitive changes; however, evidence is mixed and confounded.	May reduce systemic anesthetic and opioid exposure, but direct evidence for better POCD outcomes is inconsistent.	Evidence is mixed, with no consistent long-term cognitive advantage over GA.
Neuroinflammation/stress response	Surgical stress and anesthetic exposure may contribute to inflammation, depending on agent, dose, depth, and patient vulnerability.	May reduce surgical stress and opioid exposure, but cognitive effects are indirect.	May reduce opioid needs and stress response, but hypotension and patient selection affect outcomes.
Hemodynamic considerations	Requires careful titration to avoid hypotension, excessive depth, and cerebral hypoperfusion.	Effects depend on block type, sedation, procedure, and comorbidities.	Provides effective anesthesia/analgesia but may cause hypotension requiring management.
Evidence limitations	Studies vary in cognitive tests, timing, surgery type, anesthetic depth, and perioperative care.	Often studied within multimodal analgesia, making isolated cognitive effects difficult to assess.	Mostly studied in selected surgeries, especially orthopedic procedures; generalizability is limited.

GA, general anesthesia; POCD, postoperative cognitive dysfunction.

**Table 3:** Major risk factors associated with POCD in elderly surgical patients.

Category	Risk Factors
Patient-related factors	Advancing age [35], pre-existing mild cognitive impairment [36], neuroinflammatory conditions, diabetes mellitus [37], elevated alkaline phosphatase levels [38], vitamin D deficiency [39], elevated beta-amyloid levels [40]
Surgery-related factors	Cardiac surgery in elderly patients [41], TKA, and THA [42], pelvic organ prolapse surgery in older women [43], hyperperfusion, degree of ICA stenosis, cerebral small vessel disease [44], laparoscopic surgery [45]
Anesthesia-related factors	Polypharmacy [46], benzodiazepine use, prolonged duration of surgery [45], prolonged preoperative hospitalization [47]
Other contributing factors	Genetic predisposition, alterations in microglial morphology [48]

POCD—Postoperative Cognitive Dysfunction; ALP—Alkaline Phosphatase; TKA—Total Knee Arthroplasty; THA—Total Hip Arthroplasty; ICA—Internal Carotid Artery.

## Data Availability

All authors thank the University of Oradea for providing the logistic facilities they used.
